# RNA Folding Energy of Long-Range Genomic Interactions Regulates Discontinuous Transcription in SARS-CoV-2

**DOI:** 10.3390/v18060620

**Published:** 2026-05-29

**Authors:** Stephen J. Ross, Chengjin Ye, Simon Moxon, Elke Mühlberger, Luis Martinez-Sobrido, Daniel Cifuentes

**Affiliations:** 1 Department of Virology, Immunology, & Microbiology, Boston University Chobanian and Avedisian School of Medicine, Boston, MA 02118, USA; 2 National Emerging Infectious Diseases Laboratories, Boston University, Boston, MA 02118, USA; 3 Department of Biochemistry & Cell Biology, Boston University Chobanian and Avedisian School of Medicine, Boston, MA 02118, USA; 4 Texas Biomedical Research Institute, San Antonio, TX 78227, USA; 5 School of Biological Sciences, University of East Anglia, Norwich NR4 7TJ, UK

**Keywords:** SARS-CoV-2, MHV, coronavirus, discontinuous transcription, RNA–RNA interactions, folding energy

## Abstract

Coronaviruses use discontinuous transcription to generate subgenomic RNAs (sgRNAs) that encode structural and accessory proteins. However, the factors regulating sgRNA abundance in SARS-CoV-2 remain unclear. Here, we combined strand-specific RNA sequencing, RNA–RNA interaction mapping, prediction of RNA folding energies, and targeted mutagenesis to define the regulation of (–) sgRNA synthesis in SARS-CoV-2 infection. We demonstrated that the relative (–) sgRNA abundance across viral genes is stable throughout infection and largely correlates with corresponding (+) sgmRNA levels. Through meta-analysis of published SPLASH data, we found that the frequency of long-range interactions between the 5′ genomic transcription regulatory sequence TRS-Leader and downstream TRS-Body sequences correlates with sgRNA abundance. Notably, the folding energy (ΔG) of these duplexes quantitatively predicts (–) sgRNA transcript levels. Mutations in non-coding regulatory regions that altered the ΔG resulted in corresponding changes in (–) sgRNA expression, suggesting a causal role for TRS duplex stability in transcriptional regulation. Analysis of naturally occurring mutations near regulatory sites further suggests that modulation of duplex stability may also serve as an evolutionary mechanism to fine-tune viral gene expression. Together, our findings identify the pairing stability of TRS-Leader:TRS-Body as a determinant of discontinuous transcription and reveal how RNA pairing potential contributes to the regulation of (–) sgRNA synthesis in SARS-CoV-2.

## 1. Importance

Viruses must carefully control how much of each of their genes they express to replicate efficiently. In this study, we asked how some coronavirus genes consistently produce more mRNA than others during infection. By combining multiple RNA sequencing approaches, analysis of RNA–RNA interactions, and targeted viral mutations, we uncovered a simple and quantitative explanation for this behavior in SARS-CoV-2.

We found that the virus relies on direct pairing between distant regions of its genome to generate the shorter RNAs needed to make most viral proteins. Crucially, the stability of these RNA pairings predicts how much RNA is produced from each gene. Stronger pairings lead to higher RNA production, while weaker pairings reduce it. When we experimentally altered these pairing strengths, RNA output changed in the expected direction, demonstrating that this mechanism directly controls gene expression.

Together, our results show that SARS-CoV-2, like other coronaviruses, encodes the determinants of gene expression levels directly in its RNA interactions, revealing a fundamental principle of coronavirus transcription.

## 2. Introduction

Coronaviruses are positive-sense, single-stranded RNA viruses with large genomes organized into two functional regions. The 5′ two-thirds of the genome encode the pp1a and pp1ab polyproteins, which are translated directly from the incoming genomic RNA and processed into the viral replicase complex [1,2,3,4]. In contrast, the 3′ portion of the genome encodes structural and accessory proteins, which are expressed through a unique transcriptional mechanism known as discontinuous transcription [3,5]. This process produces a nested set of negative-sense (–) subgenomic RNAs (sgRNAs), each serving as a template for the synthesis of a corresponding positive-sense (+) subgenomic mRNA (sgmRNA) that drives protein production [3,6].

**Figure 1:**
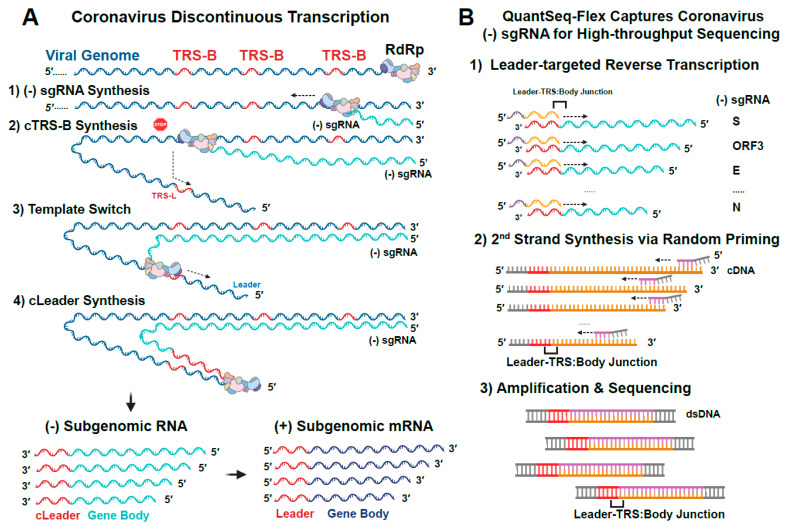
Schematics of discontinuous transcription and QuantSeq-Flex methodology. (**A**) (1) The viral RNA-dependent RNA polymerase (RdRp) complex binds to the 3’ end of the genome (blue) to initiate transcription. (2) The RdRp begins RNA synthesis for a nascent strand of RNA complementary to the genome (teal). (3) Once RdRp engages with the core sequence of a given TRS-B (red), a template switching event occurs, allowing RdRp to ‘jump’ to the leader sequence at the 5’ end of the genome at the TRS-L (red). (4) The viral RdRp continues RNA synthesis until it reaches the 5’ genomic end. Discontinuous transcription results in the production of a set of nested (–) sgRNAs (teal) that all contain the same sequence complementary to the (+) gRNA leader (cLeader) at their 3’ end (shown in red box). The viral RdRp utilizes these (–) sgRNAs as templates for a second round of transcription to synthesize complementary (+) sgmRNAs (blue). These (+) sgmRNAs are the final product of coronavirus transcription and are translated as viral structural and accessory proteins. (**B**) QUANT-Seq Flex protocol for quantifying coronavirus (–) sgRNAs. (1) Total cellular RNA isolated from coronavirus-infected cells was used as a template to synthesize the first strand (cDNA) of the (–) sgRNA library. A primer that is identical to the viral (+) gRNA leader sequence was utilized to capture all (–) sgRNAs expressed during first strand synthesis (orange). Placing the primer within the leader sequence allows for the generation of cDNA containing the TRS-L:TRS-B junction necessary for the identification of each individual (–) sgRNA. This junction acts as a unique barcode for the in silico quantification of each (–) sgRNA species during subsequent sequencing analysis. (2) A proprietary mix of random primers is used for second strand synthesis to create a dsDNA library. (3) After library amplification and purification, this pipeline results in the generation of dsDNA libraries each containing a TRS-L:TRS-B junction specific to each individual (–) sgRNA. (–) sgRNAs are quantified through strand-specific mapping (hisat2) and TRS-L:TRS-B junction extraction using regtools, a software commonly used for extracting differential splice sites (junctions) in RNA sequencing datasets.

Discontinuous transcription is mediated by the viral RNA-dependent RNA polymerase (RdRp), which initiates RNA synthesis from the 3′ end of the genome. Upon encountering a transcription regulatory sequence (TRS) in the body of the genome (TRS-B), the polymerase can pause and switch templates by base-pairing with a complementary sequence at the 5′ genomic end, known as the TRS-Leader (TRS-L) [5,6]. This template switch generates (–) sgRNAs that share a common 3′ leader sequence [7]. These serve as templates for the production of (+) sgmRNAs, which are translated into structural and accessory proteins essential for virion assembly (Figure 1A) [3].

Although all TRS-Bs contain a conserved core sequence (CS) of 6–7 nucleotides [8], (–) sgRNAs are produced in gene-specific, reproducible ratios, raising a fundamental question: How does the RdRp distinguish between gene-specific TRSs to achieve such finely tuned transcript levels? While deletion of the CS eliminates (–) sgRNA production, variation in the flanking nucleotides modulates transcript abundance by several fold, suggesting that structural or sequence elements beyond the CS influence transcription efficiency [9,10,11].

Studies in transmissible gastroenteritis virus (TGEV) and other coronaviruses have implicated RNA–RNA interactions between TRS-L and TRS-B as facilitators of template switching. Both short- and long-range interactions have been proposed to stabilize the RdRp complex during transcription. With TGEV, the thermodynamic stability (ΔG) of TRS-L:TRS-B duplexes was shown to correlate with the expression of corresponding (+) sgmRNAs [9]. While similar long-range RNA structures have been observed in SARS-CoV-2, the functional significance of these interactions in governing discontinuous transcription remains unclear [12,13,14,15]. Moreover, previous studies primarily quantified the abundance of (+) sgmRNAs, which are not the direct product of the discontinuous transcription process itself, and their levels could also be influenced by mRNA degradation. In contrast, (–) sgRNAs represent the direct output of discontinuous transcription and offer more accurate insights into the underlying regulatory mechanisms.

To address these gaps, specifically in SARS-CoV-2, we developed a strand-specific RNA-Seq approach to accurately quantify (–) sgRNAs (Figure 1B). We applied this method to analyze transcript stoichiometries during infection with SARS-CoV-2 (*Betacoronavirus pandemicum*), recombinant SARS-CoV-2 (rSARS-CoV-2), and recombinant murine hepatitis virus (rMHV; *Betacoronavirus muris*). Although SARS-CoV-2 and MHV are both betacoronaviruses, they belong to distinct subgenera, *Sarbecovirus* and *Embecovirus*, respectively, and represent model coronaviruses with distinct transcriptional profiles. By integrating sgRNA expression data with genome-wide RNA–RNA interaction mapping and prediction of RNA folding energies, we uncovered a direct relationship between TRS-L:TRS-B duplex stability and transcript abundance. Moreover, targeted mutations altering TRS duplex interactions resulted in predictable changes in (–) sgRNA expression of SARS-CoV-2.

Together, our findings support a model in which the stability of long-range RNA–RNA interactions encode the transcriptional logic in SARS-CoV-2, providing a mechanistic framework for understanding how relative (–) sgRNA levels are established during infection.

## 3. Materials and Methods

### 3.1. Biosafety Statement

All experiments involving infectious SARS-CoV-2 were conducted in biosafety level 3 (BSL-3) laboratories at the Texas Biomedical Research Institute (Texas Biomed) under approvals from the Texas Biomed Institutional Biosafety Committee (IBC).

### 3.2. Cell Culture

L929 cells (mouse connective tissue; ATCC CCL-1) and 17CL-1 cells (mouse fibroblasts; kindly provided by Dr. V. Thiel, Institution of Virology and Immunology, Bern, Switzerland) were maintained in Eagle’s Minimum Essential Medium (EMEM; Corning, NY, USA) supplemented with 10% fetal bovine serum (FBS; R&D Systems, Minneapolis, MN, USA), 2 mM L-glutamine (Thermo Fisher Scientific, Waltham, MA, USA), and either 100 U/mL penicillin–streptomycin (Thermo Fisher Scientific, Waltham, MA, USA) or 100 μg/mL Primocin (Invivogen, San Diego, CA, USA). African green monkey kidney epithelial cells (Vero E6, ATCC CRL-1586) were maintained in Dulbecco’s modified Eagle medium (DMEM, Corning, NY, USA) supplemented with 5% FBS (VWR) and 1% penicillin–streptomycin–glutamine (PSG, Corning, NY, USA). Cells were grown at 37 °C with 5% CO_2_.

### 3.3. Generation of Viral Stocks

For this study, we used recombinant MHV expressing GFP (MHV-GP33-GFP) kindly provided by Dr. V. Thiel, Institution of Virology and Immunology, Bern, Switzerland. In this work, this virus will be referred to as rMHV [16]. Briefly, this mutant contains the GP33 epitope and a GFP reporter gene inserted between nucleotides 27,967 and 28,700 of the wild-type (WT) MHV genome. The rMHV construct containing this insertion did not show defects in viral growth kinetics compared to WT virus [16]. rMHV was grown in 17CL-1 cells in EMEM containing 2% FBS, 2 mM L-glutamine, and 100 U/mL penicillin–streptomycin. Then, 4.7 × 10^6^ 17CL-1 cells were plated in a T175 flask (Corning, NY, USA) and inoculated with 100 µL of rMHV (P + 1). The supernatant was harvested 2 days post infection (dpi) and clarified by centrifugation at 5000× *g* for 10 min at room temperature. Clarified supernatants were aliquoted into 300 µL viral stocks.

SARS-CoV-2 was grown in Vero E6 cells cultured in DMEM supplemented with 2% FBS, 2 mM L-glutamine, and either 100 U/mL penicillin–streptomycin (Thermo Fisher Scientific, Waltham, MA, USA) or 100 mg/mL Primocin. The supernatant was harvested 2–3 dpi based on cytopathic effect (CPE) and clarified by centrifugation at 5000× *g* for 10 min at room temperature. Clarified supernatants were aliquoted into 1 mL viral stocks. Virus titers were determined in L929 cells (rMHV) or Vero E6 cells (SARS-CoV-2) either by 50% tissue culture infectious dose (TCID_50_) assay and calculated using the Spearman–Kärber algorithm or by plaque assay.

### 3.4. Generation of rSARS-CoV-2

The rSARS-CoV-2 WT and mutant viruses used in this study were generated using the bacterial artificial chromosome (BAC)-based reverse genetic system (see Table 1) [17]. The rSARS-CoV-2 WT sequence is based on the SARS-CoV-2 Washington strain (WA1, GenBank: MN985325.1). For the mutant rSARS-CoV-2, mutations were introduced into the intermediate plasmid (pUC57-F1) using site-directed mutagenesis (SDM). The modified F1 fragment was then excised via BsmBI digestion and used to replace the corresponding region in the BAC backbone, following the previously described protocol [18]. The mutant rSARS-CoV-2 was recovered by transfection of Vero E6 cells with the resultant BAC as described previously [18].

### 3.5. SARS-CoV-2 Infection

Monolayers of Vero E6 cells (6-well format, 1 × 10^6^ cells, triplicates) were infected with rSARS-CoV-2 at a multiplicity of infection (MOI) of 10. Following a 1 h viral adsorption at 37 °C with 5% CO_2_, the inoculum was removed and replaced with 2 mL per well of post-infection medium. At 24 h post-infection (hpi), the supernatant was discarded, and the cells were lysed in TRIzol reagent (1 mL/well, Thermo Fisher Scientific, Waltham, MA, USA) according to approved institutional inactivation protocols. The inactivated samples were then removed from the BSL-3 laboratory, and total cellular RNA was extracted for further analysis.

### 3.6. rMHV Infection

L929 cells (6-well plates, 5 × 10^5^ cells/well) were infected with rMHV at an MOI of 5 in a volume of 500 μL. Infections were performed in triplicate wells. To synchronize infection, plates were covered with parafilm immediately after the addition of inoculum and placed in a 16 °C water bath. After 1 h, 1.5 mL of EMEM pre-warmed to 37 °C was added to each well. Plates were placed at 37 °C, 5% CO_2_ until the time of collection. Cells were lysed in 1 mL of TRIzol per well at 4 h, 6 h, and 12 h post-infection. Total cellular RNA was isolated and used for both (–) sgRNA and (+) sgmRNA library generation.

### 3.7. Poly(A)-Selected RNA-Seq

An amount of 10 μg of total RNA isolated from rMHV-infected cells was treated with DNase I (NEB, Ipswich, MA, USA) according to the manufacturer’s instructions. Total RNA was isolated from the DNase I reaction via acid phenol:chloroform extraction and isopropanol precipitation. Briefly, equal parts of acid phenol and chloroform were added to the DNase I reaction. Tubes were placed on ice and centrifuged at ~12,000× *g* at 4 °C in a table-top centrifuge. The aqueous phase was transferred to a new tube and precipitated with 40 μL 3 M sodium acetate, 12 μL glycerol, and 1 mL of 100% isopropanol for 1 h at −20 °C. Tubes were centrifuged for 10 min at ~20,000× *g*. The pellets were washed with 70% ethanol and resuspended in 20 μL of nuclease free H_2_O. RNA was incubated at 55 °C for 15 min for homogenization. An amount of 1 µg of DNase I treated RNA was spiked with 50 ng of yeast RNA. Samples were submitted to Boston University (BU) Sequencing Core for poly(A) selection, library preparation, and sequencing.

### 3.8. QuantSeq-Flex (–) sgRNA RNA-Seq Library Preparation

An amount of 300 ng to 1 µg of total cellular RNA from rMHV-infected L929 cells or Vero E6 cells infected with rSARS-CoV-2 WT, rSARS-CoV-2 ΔORF8, rSARS-CoV-2 ΔORF7a, or rSARS-CoV-2 ORF8 ΔG was used as a template for reverse transcription and (–) sgRNA library generation using the QuantSeq-Flex kit (Lexogen Inc., Greenland, NH, USA). Libraries were created according to the manufacturer’s instructions. Briefly, primers (see below) which bind to nucleotides 26 to 48 of the SARS-CoV-2 or nucleotides 35 to 54 of the rMHV (–) sgRNA 3′ end leader regions, respectively, were used for first strand synthesis. The template RNA was degraded prior to second strand synthesis using reagents provided in the kit. Libraries were amplified via PCR using QuantSeq-Flex kit reagents. Libraries at a final concentration of 1–2 ng DNA/μL were sent to the Yale Center for Genomic Analysis (YCGA) for sequencing.

SARS-CoV-2 primer:

5’ GTTCAGACGTGTGCTCTTCCGATCTTAACAAACCAACCAACTTTCGATC 3’

rMHV primer:

5’ GTTCAGACGTGTGCTCTTCCGATCTTCAACTCTAAAACTCTTGTAG 3’

### 3.9. Yeast RNA Extraction

A 5 mL culture of *Saccharomyces cerevisiae* strain VL6-48N was used for total RNA extraction. Cells were briefly centrifuged, and the cell pellet was resuspended in 1 mL of ice-cold water. The cells were briefly centrifuged again, and the supernatant was removed. The resulting cell pellet was frozen on dry ice and thawed just before continuing the protocol. The cell pellet was resuspended in 400 μL TES solution (10 mM Tris HCl, pH 7.5, 10 mM EDTA, 0.5% SDS). Then, 400 μL of acid phenol was added to the solution, and the tube was vortexed vigorously for 10 s. The tubes were incubated for 60 min at 65 °C with occasional, brief vortexing. An amount of 400 μL chloroform was added and each tube was vortexed vigorously and centrifuged at 12,000× *g* for 5 min at 4 °C. The aqueous phase was transferred to a new tube with 40 μL of 3 M sodium acetate, pH 5.3, 12 μL of glycerol, and 1 mL of ice-cold 100% ethanol, and incubated for 60 min to precipitate the RNA. RNA was pelleted in a microcentrifuge for 5 min at 20,000× *g* at 4 °C. The pellet was washed with 1 mL of 70% ethanol and resuspended in 20 μL of nuclease free H_2_O.

### 3.10. In Silico TRS ΔG Analysis

Free energy (ΔG) values for TRS-L:TRS-B interactions were calculated using the ViennaRNA RNAcofold algorithm. Briefly, the rMHV or rSARS-CoV-2 TRS-L and the following 3’ 10 nucleotides were used to predict binding with each complementary (c) TRS-B and following 3’ 10 nucleotides. These sequences were used as input into the ViennaRNA RNAcofold software version 2.6 at http://rna.tbi.univie.ac.at/cgi-bin/RNAWebSuite/RNAcofold.cgi (accessed on 8 March 2022). Free energy calculations were produced by the software using default settings.

### 3.11. sgRNA Quantification

FASTQ files obtained from BU, YCGA, or Sequence Read Archive (SRA) available datasets (see Table 2) were trimmed for Illumina adaptor content using Cutadapt. Trimmed FASTQ files were mapped to the MHV-GP33-GFP [16] or SARS-CoV-2 genome (MN985325) using Hisat2 v2.2.1. Once mapped, .sam and .bam files were processed using samtools. Junction reads were quantified using regtools (-junctions extract) with sorted and indexed .bam files. The resulting .bed files were used to obtain raw chimeric read counts. Reads were normalized by total chimeric reads. Statistical analyses were performed using GraphPad Prism v10.

## 4. Results

### 4.1. Coronavirus (–) sgRNA Expression Determines (+) sgmRNA Abundance

Coronaviruses capitalize on discontinuous transcription to efficiently generate a nested set of (–) sgRNAs that drive the expression of multiple structural and accessory proteins. This process involves the viral RdRp jumping between genome regions (template switching) during negative-strand synthesis, guided by transcription regulatory sequences (TRSs). To uncover the molecular features that determine the frequency of template switching during coronavirus transcription, previous studies on discontinuous transcription primarily focused on (+) sgmRNA expression. However, because (+) sgmRNAs are not the direct product of discontinuous transcription, additional factors like mRNA degradation may influence their abundance, potentially confounding analyses of the actual frequency of template switching.

Therefore, we focused our analysis on the quantification of (–) sgRNA, the direct product of template switching, as a proxy for the relative frequency of template switching. Traditionally, (–) sgRNA quantification has relied on Northern blot analysis of individual viral genes. Here, we aimed to investigate the relative abundance of (–) sgRNA using a highly quantitative, high-throughput approach based on next-generation sequencing (NGS). However, direct quantification of (–) sgRNA cannot be achieved using standard RNA-Seq techniques because (–) sgRNAs lack a poly(A) tail. Additionally, the nested nature of (–) sgRNAs results in sequence overlap, making it difficult to assign short sequencing reads to specific (–) sgRNA species.

To address these challenges, we adapted QuantSeq-Flex to specifically sequence the template-switching boundary, the TRS-Leader:TRS-Body junction, and to quantify all (–) sgRNAs species produced during infection. QuantSeq-Flex capitalizes on the fact that all (–) sgRNA species share an identical 3′ end. We used a single oligonucleotide complementary to this conserved 3′ end to prime the reverse transcription of (–) sgRNA into cDNA (Figure 1B). The second strand was then synthesized using random nucleotides, simultaneously incorporating a fixed adaptor sequence. Amplification using primers targeting this adaptor and the conserved leader sequence generated a sequencing-ready library, where each diagnostic read contained a TRS-L:TRS-B junction. The sequencing coverage at each TRS-L:TRS-B junction provides the basis for the precise quantification of the relative abundance of (–) sgRNAs (Figure 1B).

**Figure 2:**
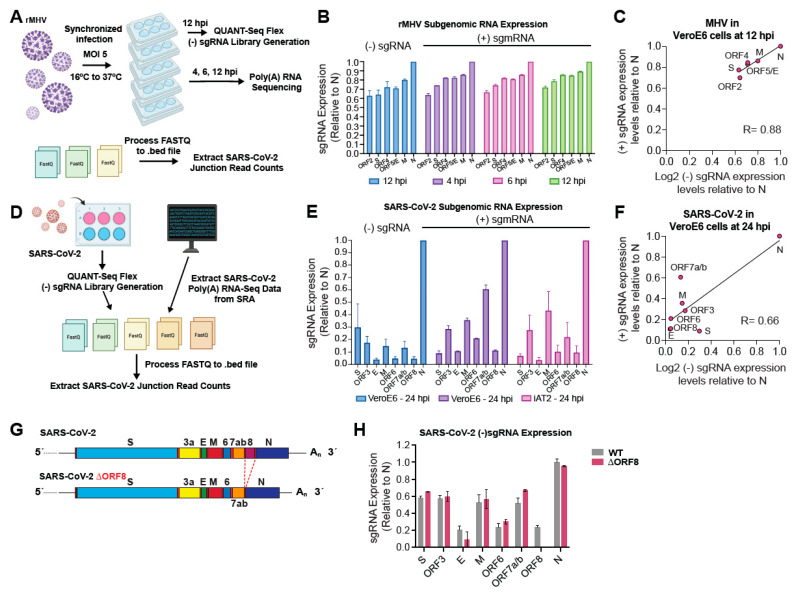
Coronavirus (–) sgRNA production correlates with (+) sgmRNA expression. (**A**) Schematic depicting rMHV infection and viral RNA library preparation: 1 × 10^6^ L929 cells were infected with rMHV at an MOI of 5. For (–) sgRNA analysis, total RNA was harvested from infected cells 12 h post infection (hpi) and used for (–) sgRNA library cloning. For (+) sgmRNA analysis, total RNA was extracted from infected cells 4, 6, and 12 hpi and used for poly(A) selected library cloning and sequencing. (**B**) Read counts from both (–) sgRNA and poly(A)-selected sequencing from (A) were used to normalize (–) sgRNA expression to N transcript expression levels (N read counts set to 1) for comparison across all datasets. Data derived from biological duplicates. (**C**) Correlation of (–) sgRNA and (+) sgmRNA of MHV in VeroE6 cells at 12 hpi, data extracted from (**B**). (**D**) 1 × 10^6^ Vero E6 cells were infected with SARS-CoV-2 at an MOI of 10. 24 hpi, cells were harvested in TRIzol, and 500 ng to 1 µg of total cellular RNA was used for (–) sgRNA library preparation. (+) sgmRNA abundance was calculated using published SARS-CoV-2 RNA-Seq datasets. Reads corresponding to TRS-B:Leader sequence junctions for all datasets were quantified and normalized to total chimeric reads. (**E**) Read counts from both (–) sgRNA and re-analyzed published poly(A)-selected sequencing datasets from (**D**) were used to normalize (–) sgRNA expression to N transcript expression levels (N read counts set to 1) for comparison across all datasets. Plots are colored according to the cell type of each dataset analyzed and the time of sample collection. Data derived from biological duplicates. (**F**) Correlation of (–) sgRNA and (+) sgmRNA of SARS-CoV-2 in VeroE6 cells at 24 hpi, data extracted from (**E**). (**G**) Schematics of SARS-CoV-2 structural and accessory genes (top) and rSARS-CoV-2 ΔORF8 (bottom). All genes were kept intact while the CDS of ORF8 was removed from the WT SARS-CoV-2 genome. (**H**) 1 × 10^6^ Vero E6 cells were infected with WT (pink) or rSARS-CoV-2 ΔORF8 mutant (black) at an MOI of 10 and total RNA was harvested 24 hpi for (–) sgRNA library generation. The graph is shown in fragments per kilobase (FPK) of TRS-L:TRS-B junction-spanning reads. Data derived from biological duplicates.

To determine the relative abundance of (–) sgRNAs across the viral genome, we quantified (–) sgRNA levels from a recombinant mouse hepatitis virus containing a GFP reporter, referred to as rMHV [16]. MHV, which belongs to the embecovirus subgenus within the betacoronavirus genus, serves as an ideal model for studying discontinuous transcription due to its well-characterized transcriptional program, genetic tractability, and shared replication mechanisms with other coronaviruses [19]. Its robust (–) sgRNA production and compatibility with BSL-2 conditions make it both effective and accessible for mechanistic studies. L929 cells were infected with rMHV at an MOI of 5 (Figure 2A). Total RNA was extracted and processed for RNA sequencing of rMHV (–) sgRNAs using the QuantSeq-Flex protocol (Figure 1B).

Quantification of rMHV (–) sgRNA abundance revealed a 3′ to 5′ expression gradient across the viral genome at 12 hpi. Specifically, genes positioned toward the 5′ end of the genome exhibited lower expression levels than those at the 3′ end (Figure 2B). MHV N (–) sgRNA transcript was the most abundant, whereas ORF2 was the least expressed.

Next, we measured (+) sgmRNA levels using standard poly(A)-selected RNA-Seq. Because (+) sgmRNAs are synthesized from (–) sgRNA templates rather than being directly transcribed from the genome, we hypothesized that their abundance would be proportional to (–) sgRNA levels, if no additional cellular mechanisms modulate transcription or induce degradation. Our data confirmed this hypothesis as (+) sgmRNA levels followed the same 3′ to 5′ expression gradient as their corresponding (–) sgRNA templates. This finding aligns with previous reports of MHV (+) sgmRNA expression [20] and validates our transcript quantification method. The 3′ to 5′ expression gradient was observed as early as 4 hpi and remained stable at 6 and 12 hpi (Figure 2B, Supplementary Figure S1). These results establish a direct correlation between (–) sgRNA and (+) sgmRNA expression, indicating that (–) sgRNA abundance is the main determinant of (+) sgmRNA levels during MHV infection (Figure 2C). Albeit, we did not test the impact of RNA degradation in the final output, our results suggest that if any degradation then it should affect both RNA species equally.

To determine whether the pattern of (–) sgRNA expression correlates with (+) sgmRNA expression across different coronaviruses, we expanded our analysis to the betacoronavirus SARS-CoV-2 (subgenus: sarbecovirus). Using QuantSeq-Flex, we quantified (–) sgRNA abundance in Vero E6 cells infected with WT rSARS-CoV-2 at an MOI of 10 (Figure 2D). Cells were harvested at 24 hpi, and total RNA was extracted for (–) sgRNA library preparation.

Unlike the 3′ to 5′ gradient observed for MHV (–) sgRNAs, SARS-CoV-2 (–) sgRNAs exhibited an alternating pattern of high and low expression across genes (Figure 2E). Specifically, ORF3a, M, and ORF7ab (–) sgRNAs were highly expressed, whereas E, ORF6, and ORF8 (–) sgRNAs showed lower expression levels. This distinct pattern suggests that SARS-CoV-2 transcription might be regulated by factors beyond genome organization alone.

To further assess whether SARS-CoV-2 (–) sgRNA and (+) sgmRNA expression levels correlate, we analyzed publicly available poly(A)-RNA sequencing datasets from SARS-CoV-2 infections in diverse cell types, including Vero E6, Calu-3, Caco-2, A549-hACE2 (A549 cells expressing human angiotensin converting enzyme 2), and induced pluripotent stem cell-derived alveolar epithelial type 2 (iAT2) cells, at various MOIs and time points (Table 2, Figure 2D), [21,22,23,24]. To enable cross-dataset comparisons, all (–) sgRNA species were normalized to N transcript levels (Figure 2E, Supplementary Figure S1). Across datasets, SARS-CoV-2 (+) sgmRNA levels were generally proportional to their corresponding (–) sgRNA templates. Two notable exceptions were observed: the S and ORF7a/b genes deviated from the overall correlation between (–) sgRNA and (+) sgmRNA levels in Vero E6 cells at 24 hpi (Figure 2F). These outliers suggest that gene-specific transcriptional regulation and/or post-transcriptional processes contribute to SARS-CoV-2 RNA abundance (Figure 2F). For example, differences in polyA tail length could bias the enrichment of specific (+) sgmRNA during polyA-dependent capture for RNA-Seq.

In contrast to MHV, SARS-CoV-2 (–) sgRNAs did not follow a 3′ and 5′ gradient. To explore the role of gene order in SARS-CoV-2 transcription, we generated a rSARS-CoV-2 mutant lacking the ORF8 gene, including the corresponding TRS-B, to assess changes in (–) sgRNA expression (Table 1, Figure 2G). Removing the ORF8 gene changes the position of the upstream located genes relative to the 3′ end of the genome, where the viral RdRp initiates transcription. If polymerase disengagement increased with distance, then rearranging the gene order should affect (–) sgRNA expression levels. Vero E6 cells were infected with WT or ΔORF8 rSARS-CoV-2 viruses, and total RNA was harvested at 24 hpi for (–) sgRNA library generation. Our results show that transcript expression remained largely unchanged in the ΔORF8 mutant compared to the WT virus (Figure 2H), confirming that the relative distance of TRS-B to the 3′ end of the genome template does not significantly impact SARS-CoV-2 (–) sgRNA abundance. Since the removal of ORF8 did not significantly alter the relative expression of other SARS-CoV-2 structural or accessory (–) sgRNAs, this suggests that each gene is transcribed separately, rather than being part of a coordinated expression cascade starting at the 3′ end of the genome.

Comparison of the murine hepatitis virus (MHV) 3′–5′ expression gradient with the alternating high- and low-expression pattern observed in SARS-CoV-2, combined with deletion analysis, demonstrated that (–) sgRNA abundance is uncoupled from the order of genes in the genome, whereas (+) sgmRNA levels are largely determined by their corresponding (–) sgRNA templates. This proportional relationship was maintained across multiple cell types, multiplicities of infection, and infection time points, indicating a conserved regulatory feature. Notably, correlations between (–) sgRNA and (+) sgmRNA expression levels were established early during infection and remained stable over time.

### 4.2. Long-Range RNA–RNA Interactions Predict sgRNA Transcript Abundance

The observation that (–) sgRNA abundance is not correlated with gene order in the genome suggests that additional regulatory elements must govern their expression. After characterizing the (–) sgRNA expression landscape during SARS-CoV-2 infection, we aimed to identify the key determinants driving (–) sgRNA abundance and to understand their role in transcriptional regulation.

**Figure 3:**
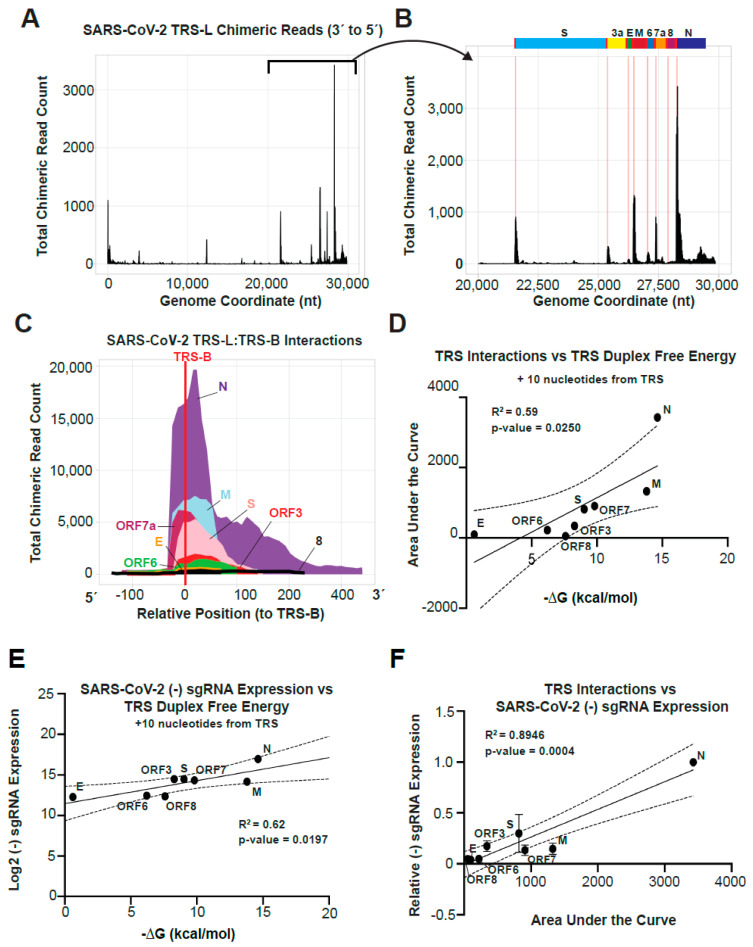
Long-range intragenomic RNA–RNA interactions reveal determinants of (–) sgRNA expression. (**A**) 3’ to 5’ chimeric read counts were filtered to show only those containing nucleotides 1–100 of the TRS-L sequence. The graph shows the 5’ arm of the chimeric reads to illustrate which sequences were ligated to the TRS-L during the SPLASH protocol. Shown are the total chimeric read counts (y-axis) across the SARS-CoV-2 genome (x-axis). This graph depicts which region of the genome interactions occurs with the first 100 nucleotides of the leader sequence. (**B**) Data from (**A**) showing only nucleotides 20,000 to 30,000. Red lines depict each TRS-B. SARS-CoV-2 genome (above) illustrates where the TRS-Bs and peaks of interactions lie in relation to the encoded genes. (**C**) Metaplot of each peak of genomic interactions as chimeric reads overlayed on top of one another fixed by their relation to the TRS-B (shown as 0 on the x-axis). The x-axis shows nucleotide position in relation to the TRS-B in a 5’ to 3’ manner. Reads were binned for every 10 nucleotides of the leader sequence. (**D**) Total chimeric reads from the peaks at each TRS-B (from (**B**)) were summed. Regression analysis of area under the curve (cumulative read values) and TRS duplex ΔG (+/− 10 nucleotides from TRS CS) were performed to calculate the correlation coefficient by linear regression in GraphPad Prism. (**E**) Regression analysis of (–) sgRNA expression (shown as a ratio to N expression) and TRS duplex ΔG was performed by linear regression in GraphPad Prism. (**F**) Total chimeric reads from the peaks at each TRS-B (from (**C**), data from (**D**)) were summed. Regression analysis of (–) sgRNA expression and area under the curve values was performed by linear regression in GraphPad Prism 10.2.3.

Previous studies of individual genes in TGEV and arteriviruses indicated that both short- and long-range RNA–RNA interactions contribute to (+) sgmRNA abundance and template switching [11,25]. To test whether analogous mechanisms regulate (–) sgRNA expression in SARS-CoV-2, we applied a high-throughput genomics approach, analyzing published SPLASH datasets (psoralen crosslinked, ligated, and selected hybrids) [14] (Figure 3A) to map long-range RNA–RNA interactions across the viral genome. This technique is designed to crosslink double-stranded RNA (dsRNA) regions and selectively clone those derived from intramolecular interactions. SPLASH captures dsRNA structures, enabling the identification of regions that frequently engage in intragenomic base-pairing, as reflected by high read coverage. While its resolution does not permit precise mapping of interacting nucleotides, the resulting peaks provide an integrated view of multiple interaction sites across the genome. Given that TRS-L:TRS-B interactions have been linked to (+) sgmRNA expression in TGEV, we focused our analysis on interactions between the first 100 nucleotides of the SARS-CoV-2 5′ genome end (which includes the TRS-L) and all other regions of the genome (Figure 3B). While ORF1 and ORF2 were mostly depleted of peaks, the highest coverage was observed in regions encompassing the TRS-B sequences that precede each structural and accessory gene (Figure 3C). These findings suggest that the (+)-strand genomic RNA (gRNA) folds into long-range structures that bring the TRS-L into proximity with TRS-B sites.

The strength of long-range RNA–RNA interactions is determined in part by RNA folding energy, measured as ΔG. Previous studies on other coronaviruses implicated RNA folding thermodynamics as a determinant of (+) sgmRNA expression [21]. In conjunction with the identified long-range RNA–RNA interactions (Figure 3A), we investigated whether this principle also applies to (–) sgRNA synthesis from the MHV and SARS-CoV-2 genomes. We calculated the predicted ΔG values for TRS-L:complementary TRS-B (cTRS-B) interactions for rMHV and SARS-CoV-2, using +10 nucleotides 3′ of each TRS CS (the core of each peak of interaction, Figure 3C) following previously described methods [21]. Analysis of the predicted ΔG values for sequences flanking the rMHV TRS-B sites revealed a profile with decreasing duplex stability from 3′ to 5′ across the genome. The TRS-L:cTRS-B interaction for the N gene, the first to be transcribed, showed the highest stability (plotted as −ΔG for ease of visualization), while downstream genes exhibited progressively weaker interactions. This gradient of folding energy closely mirrors the expression pattern of rMHV (–) sgRNAs and (+) sgmRNAs (Supplementary Figure S2A,B). A regression analysis confirmed a correlation between rMHV TRS duplex −ΔG and (–) sgRNA expression, with an R^2^ of 0.83 and a *p*-value of 0.0112 (Supplementary Figure S2C).

We then applied the same analysis to the SARS-CoV-2 genome. Similarly, the predicted ΔG of TRS-L:cTRS-B interactions generally tracked with TRS-L:TRS-Body interactions, (–) sgRNA and (+) transcript expression levels with an R^2^ of 0.59 and *p*-value of 0.0250 (Figure 3D). However, one notable deviation was observed at the E gene, where the −ΔG value was relatively low, despite moderate E (–) sgRNA expression (Figure 3E). This discrepancy reduced the overall correlation between TRS duplex stability and (–) sgRNA levels, yielding an R^2^ of 0.62 and a *p*-value of 0.0197 for SARS-CoV-2.

To test whether these interactions correlate with transcriptional output, we performed a regression analysis comparing (–) sgRNA expression levels with the cumulative SPLASH read coverage at each TRS-B region. This analysis revealed a correlation, with an R^2^ value of 0.8946 with a *p*-value of 0.0004 (Figure 3F), suggesting that TRS-L:TRS-B interaction frequency is a determinant of template switching during (–) sgRNA synthesis. This supports a direct link between long-range genomic TRS-L:TRS-B interactions and subsequent (–) sgRNA production.

### 4.3. TRS-L:cTRS-B Interactions Beyond the Core Sequence Influence (–) sgRNA Synthesis

**Figure 4:**
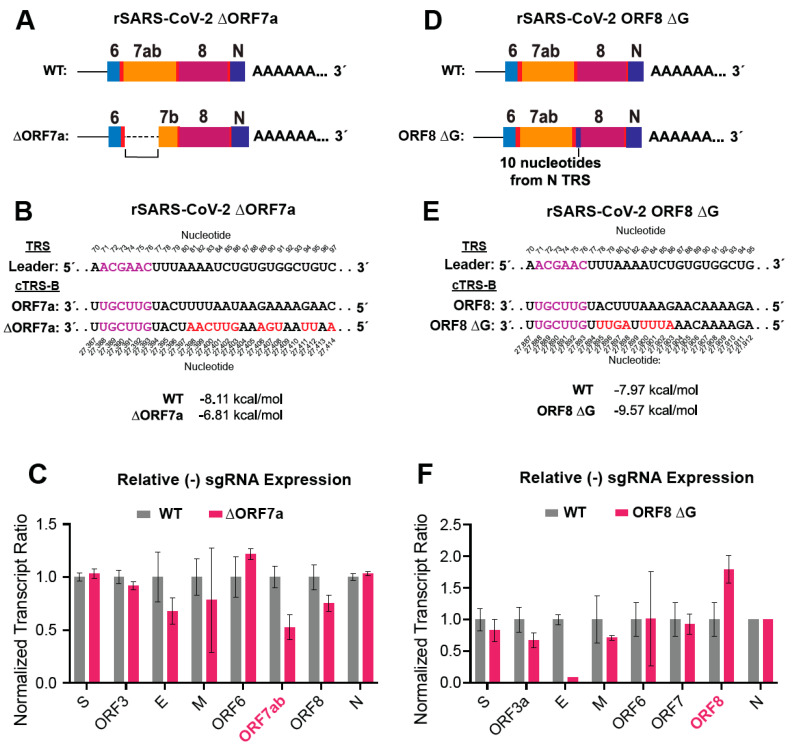
Alterations in TRS ΔG modulate (–) sgRNA expression of SARS-CoV-2 mutants. (**A**) Schematic of the rSARS-CoV-2 ΔORF7a mutant. (**B**) The WT SARS-CoV-2 genome (top) is shown in comparison to the corresponding region in the ΔORF7a mutant (bottom), with nucleotide differences shown in red. ΔG values of both WT and ΔORF7a mutant were predicted using ViennaRNA suite RNAcofold. (**C**) Analysis of rSARS-CoV-2 ΔORF7a mutant (–) sgRNA expression. Briefly, Vero E6 cells were infected with WT or mutant ΔORF7a SARS-CoV-2 and total RNA was used to clone (–) sgRNA libraries. Data are shown as chimeric read counts normalized to the wild type level of each respective gene. Data derived from biological duplicates. (**D**) Schematic of the rSARS-CoV-2 WT (top) and ORF8 ΔG mutant (bottom) viruses (**E**) WT SARS-CoV-2 genome shown in comparison to ORF8 ΔG mutant with mutations shown in red. ΔG values of both WT and ORF8 ΔG mutant were predicted using the ViennaRNA suite RNAcofold algorithm. (**F**) Analysis of rSARS-CoV-2 ORF8 ΔG mutant (–) sgRNA expression. Briefly, Vero E6 cells were infected with WT or mutant ORF8 ΔG SARS-CoV-2 and total RNA was used to clone (–) sgRNA libraries. Data are shown as chimeric read counts normalized to the wild type level of each respective gene. Data derived from biological duplicates.

To experimentally determine whether the stability of TRS-L:cTRS-B interactions modulates SARS-CoV-2 (–) sgRNA expression, we generated rSARS-CoV-2 mutants with altered nucleotide sequences immediately 3′ of select TRS-B sites on the viral genome, thereby modifying their predicted folding stabilities. The first mutant included a deletion of ORF7a, a non-essential gene with high relative (–) sgRNA expression. This deletion alters the nucleotide composition immediately downstream of the ORF7ab TRS CS (Figure 4A). In silico ΔG analysis of the altered nucleotides predicted a reduction in duplex stability with the TRS-L (Figure 4B). Consistent with this prediction, ORF7 (–) sgRNA expression was reduced compared to the WT SARS-CoV-2 (Figure 4C).

To test the inverse scenario, we engineered a second mutant to increase the predicted stability of the ORF8 TRS-B pairing with TRS-L (Figure 4D,E), where we inserted 10 nucleotides derived from the N TRS. This mutation led to an increase in ORF8 (–) sgRNA expression (Figure 4F). These results confirm that modulating the viral genome sequence 3′ of the TRS CS can tune the stability of TRS-L:cTRS-B duplex formation and directly affect (–) sgRNA transcription levels. Interestingly, in the ORF8 ΔG mutant, we also observed a reduction in E (–) sgRNA expression (Figure 2C). Although these observations suggest a potential transcriptional link between ORF8 and E, prior deletion of ORF8 did not alter E expression, indicating that any relationship is indirect. Likewise, our results support a role for duplex stability in regulating (–) sgRNA production in SARS-CoV-2 as was postulated for other coronaviruses [9,11]. However, sequence-specific contributions cannot be excluded in the absence of sequence-controlled mutants preserving ΔG. Future studies incorporating such designs will directly resolve the relative roles of duplex stability and primary sequence in TRS-mediated transcription.

In conclusion, our systematic analysis of genome-wide long-range RNA–RNA interactions, combined with targeted deletions and insertions, indicates that the stability (ΔG) of TRS-L:cTRS-B duplexes is a determinant of (–) sgRNA abundance during discontinuous transcription in SARS-CoV-2 infection. Future experiments using compensatory or ΔG-preserving sequence-scrambled mutants will be important to disentangle the contribution of duplex stability from sequence-dependent effects.

### 4.4. Evolutionary Implications of TRS Base-Pairing Potential

Our findings demonstrate that changes in the ΔG near TRS regulatory sequences modulate (–) sgRNA expression, adding to the growing recognition that RNA folding dynamics play a significant role in regulating positive-strand RNA virus transcription and replication [26,27,28,29]. This raises the question of whether manipulating TRS duplex stability represents an evolutionary strategy followed by coronaviruses to fine-tune gene expression.

**Figure 5:**
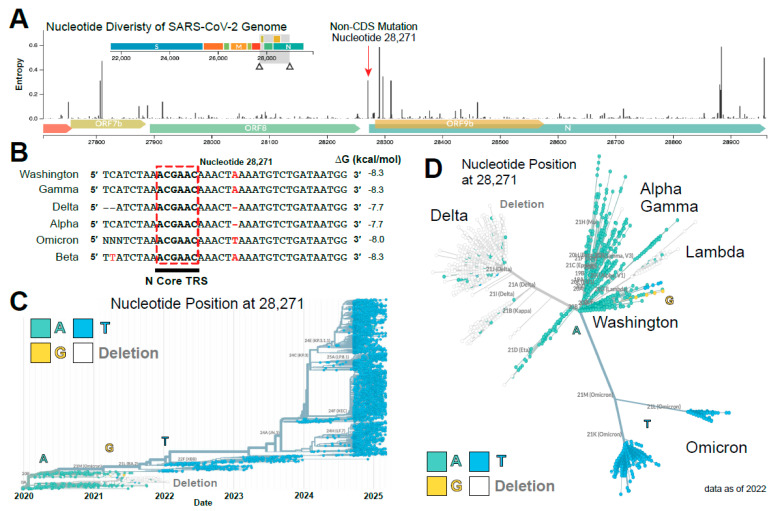
(**A**) Graph of the cumulative Shannon entropy at each nucleotide in the SARS-CoV-2 genome since 2019. Red arrow depicts nucleotide 28,271 within the N TRS. Shown is a portion of the SARS-CoV-2 structural and accessory genes as depicted in the top left of the graph (shown portion highlighted grey). (**B**) Single genome alignment of the SARS-CoV-2 N TRS. Mutations shown in red. ΔG values (kcal/mol) of each variant are listed at the right of each genome as calculated by the ViennaRNA suite RNAcofold algorithm. (**C**) Phylogenetic tree of SARS-CoV-2 variant lineages as of 2022 with each lineage colored by nucleotide composition at nucleotide 28,271. (**D**) Timeline of SARS-CoV-2 variant lineage emergence. Strains colored by nucleotide composition at nucleotide 28,271. All data from this figure were generated using the Nextstrain dataset.

To explore this hypothesis, we analyzed naturally occurring mutations near TRS sites across SARS-CoV-2 genomes. We identified the most frequently mutated non-coding position downstream of a TRS at nucleotide 28,271, located just after the TRS-B of the N gene (Shannon entropy = 0.32) (Figure 5A). At this site, three alternative nucleotides emerged: the ancestral A, found in early strains such as Wuhan (NC_045512) and WA1 (MN985325.1); a deletion, present in Delta (GISAID: EPI_ISL_6832166) variants; and a U, which is fixed in all Omicron-lineage variants (GISAID: EPI_ISL_6640916). Remarkably, this single position clearly demarcates the major SARS-CoV-2 variant lineages (Figure 5B–D). The fixation of the U variant, which results in a less stable predicted RNA duplex between TRS-L:cN-TRS-B, suggests a possible functional advantage (Figure 5B). Based on our finding that reduced TRS duplex thermodynamic stability leads to decreased (–) sgRNA production, we propose that such mutations may also reduce N protein output by impairing TRS-mediated transcription. Notably, Omicron variants harbor mutations within the N gene that generate a novel TRS, resulting in the production of a truncated N protein reported to function as a type I interferon antagonist [30]. In this context, the mutation we observe in the canonical N TRS may represent a mechanism to fine-tune N expression in the presence of this alternative TRS.

Taken together, our data suggest that modulating TRS-L:cTRS-B duplex stability provides an evolutionary mechanism for coronaviruses to regulate gene expression. Accumulation of subtle, non-coding mutations may adjust the viral transcriptomic output, thereby altering pathogenicity and shaping evolutionary trajectories.

## 5. Discussion

In this study, we integrate genome-wide quantification of (–) sgRNAs, analysis of RNA–RNA interaction frequencies, thermodynamic modeling, and targeted mutagenesis to define the determinants of discontinuous SARS-CoV-2 transcription. We show that (–) sgRNA expression levels are stable over time and directly correlate with both (+) sgmRNA abundance and the frequency of TRS-L:cTRS-B interactions. Previous studies reported a correlation between the absolute levels of (–) sgRNA and (+) sgmRNA in MHV [31,32]. Here, using QuantSeq-Flex, we refine this observation by demonstrating that this correlation is preserved at the level of individual genes rather than only at the global transcriptome scale in SARS-CoV-2.

Using SPLASH data, we identify long-range RNA–RNA interactions between TRS-L and TRS-B regions as a prominent feature of the SARS-CoV-2 genome architecture. Because SPLASH measures RNA–RNA interaction frequency rather than functional consequence, we integrated these data with RNA folding stability and targeted perturbations in recombinant viruses. This combined analysis shows that TRS-L:TRS-B duplex stability correlates with (–) sgRNA abundance and that local sequence modifications predicted to alter pairing stability can modulate transcriptional output. Together, these results support a model in which TRS-L:TRS-B pairing contributes to the regulation of coronavirus negative-strand sgRNA synthesis, while future experiments will be needed to disentangle the specific contribution of duplex stability from sequence-dependent effects.

Analysis of folding energies predicted ΔG values around the rMHV TRS-B sites that revealed a trend of decreasing duplex stability moving from the 3′ to the 5′ end of the genome. The TRS-L:cTRS-B pairing for the N gene, which is transcribed first, displays the highest stability, while the interactions associated with downstream genes become progressively weaker. This thermodynamic gradient appears to align with the expression pattern of rMHV (–) sgRNAs and (+) sgmRNAs, suggesting that duplex stability may play a role in modulating transcriptional efficiency (Supplementary Figure S2). The 3′ to 5′ gradient of rMHV (–) sgRNA expression may also be explained by an alternative hypothesis in which the viral RdRp engages in template switching at each TRS in decreasing probabilities based on the distance from the 3′ end of the genome. A 3′ to 5′ sequential reduction in TRS engagement by the RdRp might also explain the gradient of rMHV (–) sgRNA expression we observe [7]. However, similar folding energy analyses using the SARS-CoV-2 TRS sequences revealed a consistent association between TRS-L:cTRS-B stability and the expression of both (–) sgRNAs and (+) sgmRNA transcripts. The direct relationship between TRS-L:cTRS-B folding stability and the subsequent production of (–) sgRNAs demonstrated a strong correlation (Figure 3). These findings further underscore the potential regulatory role of pairing stability in governing long-range TRS-L:TRS-B interactions and influencing the transcriptional output of (–) sgRNAs in SARS-CoV-2.

Our findings of discontinuous transcription in SARS-CoV-2 build on prior studies in other coronaviruses, such as TGEV and MHV, which proposed that both RNA secondary structure and TRS complementarity contribute to (+) sgmRNA expression [33]. Previous work in TGEV suggested that base-pairing between TRS-L and complementary sequences downstream of TRS-B may facilitate template switching by stabilizing the nascent RNA strand during transcription [11,34]. Similarly, arterivirus reporter-based studies demonstrated that the strength of TRS duplex formation modulates transcription efficiency [25]. However, these studies were based on the quantification of (+) sgmRNA, instead of (–) sgRNA, the direct product of discontinuous transcription. Our study provides a genome-wide, quantitative assessment of this model in the context of SARS-CoV-2 infection. By coupling SPLASH-based interaction mapping [14] with quantitative RNA-seq of (–) sgRNA and in silico secondary structure modeling, we present direct evidence that long-range RNA–RNA interactions and their ΔG values strongly correlate with the output of each (–) sgRNA in SARS-CoV-2. Ultimately, our mutational analyses with recombinant SARS-CoV-2 establish a link between TRS duplex stability and transcript abundance, supporting the idea that RNA pairing energy is not merely correlative, but mechanistically involved in governing discontinuous transcription.

Although the resolution of SPLASH does not allow for the precise identification of the interacting nucleotides, the observed peaks likely represent the aggregate of multiple discrete interactions. Nonetheless, the SPLASH data show that the most abundant long-range interactions are concentrated at TRS-B core sequences in patterns that reflect the relative (–) sgRNA expression levels observed in wild-type rSARS-CoV-2 (Supplementary Figure S1). Combined, our analyses show that (–) sgRNA levels during SARS-CoV-2 infection correlate with subsequent (+) sgmRNA expression and that TRS-L:TRS-B interaction frequencies, as captured by SPLASH, correlate with both. These findings suggest that long-range RNA–RNA interactions are a key determinant of (–) sgRNA transcription.

Although we observed a direct correlation between TRS-L:cTRS-B duplex stability and (−) sgRNA production, these findings do not preclude the involvement of additional factors regulating coronavirus transcriptional activity that were not examined in this study, including local RNA secondary structure and host RNA-binding proteins. The coronavirus genome contains local RNA secondary structures at both the 5′ and 3′ ends that regulate multiple aspects of the viral life cycle [3,35,36]. TRS sequences themselves have also been reported to form secondary structures [13,37,38,39,40,41]. These structures may function by pausing RNA polymerization to facilitate proper TRS-L:cTRS-B pairing, implicating a potential role for coronavirus polymerase kinetics [37,42]. Other studies have shown that host RNA-binding proteins, including PTB and hnRNPA1, are required for efficient viral RNA production during MHV infection [43,44,45]. However, whether these or related host RBPs play comparable roles in SARS-CoV-2 transcription remains to be determined. Altogether, these studies highlight additional factors that may contribute to the coronavirus transcriptional mechanism.

Our findings have broader implications for understanding the evolution and regulation of coronavirus gene expression. The sensitivity of discontinuous transcription to local RNA structure and pairing potential suggests that even small sequence changes outside protein-coding regions may have a large impact on viral transcriptome architecture. Our analyses of naturally occurring SARS-CoV-2 mutations using the Nextstrain dataset revealed lineage-defining changes at non-coding positions adjacent to transcription regulatory sites. A single nucleotide substitution downstream of the nucleocapsid gene regulatory sequence, which affects predicted duplex stability, is fixed in Omicron and its descendants. These data support the idea that modulation of RNA pairing energy at regulatory elements may serve as an accessible evolutionary mechanism to adjust gene expression. Consistent with this model, certain canonical SARS-CoV-2 TRS sequences are not under purifying selection and can tolerate mutations [46]. Fine-tuning of transcriptional output through noncoding variation may therefore contribute to viral adaptation and fitness across hosts and transmission contexts. For example, changes in the SARS-CoV-2 nucleocapsid TRS can lead to the expression of a truncated nucleocapsid protein that enhances viral fitness through type I interferon antagonism [30,47]. More broadly, this work adds to the growing recognition that pairing between long-distance RNA regions is a key regulatory layer in positive-strand RNA viruses and highlights the importance of structure–function relationships beyond the protein-coding genome.

In summary, our study provides a comprehensive analysis of the mechanisms regulating coronavirus discontinuous transcription, integrating genome-wide (–) sgRNA quantification, RNA–RNA interaction profiling, RNA pairing, and functional mutagenesis. We demonstrate that the frequency of template switching is not random but instead tightly governed by the strength of TRS-L:cTRS-B interactions. These findings position RNA duplex stability as a central regulator of sgRNA synthesis in SARS-CoV-2, MHV, and likely other coronaviruses. By revealing how RNA structure encodes transcriptional logic in a segmented yet continuous genome, our work advances our understanding of coronavirus RNA biology.

## Figures and Tables

**Table 1:** List of rSARS-CoV-2 mutant viruses used in this study.

Mutant Name	Type of Mutation	Nucleotides	Description
ΔORF8	Deletion	27,888–28,259	ORF8, including its TRS, were deleted from the SARS-CoV-2 WA1 genome
ΔORF7a	Deletion	27,394–27,755	ORF7a was deleted from the SARS-CoV-2 WA1 genome, leaving TRS intact for the expression of ORF7b
ORF8 ΔG Mutant	Substitution	27,894–27,903	The first 3′ 10 nucleotides of the ORF8 TRS were substituted with nucleotides that decrease the predicted ΔG value of this sequence (to increase thermodynamic stability of predicted TRS duplex).

**Table 2:** Poly(A)-selected RNAseq datasets for SARS-CoV-2 (+) sgmRNA analysis. All listed datasets are publicly available and were downloaded from SRA (SRA link within original publication). For ease of access, for each study we included DOI, first author, virus, viral reference sequence, cells used for infection, timepoint RNA which was collected, the MOI used for initial infection, the type of sequencing performed, and the number of replicates analyzed. Once downloaded, the data were processed as described (see Methods—sgRNA Quantification). Reads corresponding to SARS-CoV-2 (+) sgmRNA transcripts were quantified for subsequent analyses.

DOI	Author	Virus	Reference	Cells	Timepoint	MOI	Sequencing	Replicates
10.1016/j.cell.2020.04.026	Blanco-Melo	SARS-CoV-2	NC_045512.2	Calu-3	24 hpi	2	poly(A)	3
10.1016/j.cell.2020.04.026	Blanco-Melo	SARS-CoV-2	NC_045512.2	A549-ACE2	24 hpi	2	poly(A)	3
10.1016/j.isci.2021.102151	Wyler	SARS-CoV-2	AY310120	Caco-2	12 hpi	0.3	poly(A)	2
10.1016/j.isci.2021.102151	Wyler	SARS-CoV-2	AY310120	Caco-2	24 hpi	0.3	poly(A)	2
10.1038/s41586-020-2739-1	Finkel	SARS-CoV-2	NC_045512.2	Vero E6	5 hpi	0.2	poly(A)	2
10.1038/s41586-020-2739-1	Finkel	SARS-Cov-2	NC_045512.2	Vero E6	24 hpi	0.2	poly(A)	2
10.1016/j.stem.2020.09.013	Huang	SARS-Cov-2	NC_045512.2	iAT2	24 hpi	5	Bulk RNA	3
10.1016/j.stem.2020.09.013	Huang	SARS-Cov-2	NC_045512.2	iAT2	96 hpi	5	Bulk RNA	3

## Data Availability

Sequencing files will be deposited in Genbank. All Illumina sequencing data for this manuscript is deposited under the accession number GSE295329. Link to data: https://www.ncbi.nlm.nih.gov/geo/query/acc.cgi?acc=GSE295329 (accessed on 21 April 2026). All SPLASH data used for analysis can be found with the accession number GSE164565 (https://www.ncbi.nlm.nih.gov/geo/query/acc.cgi?acc=GSE164565 (accessed on 28 September 2022)).

## Supplementary Materials

The following supporting information can be downloaded at: https://www.mdpi.com/article/10.3390/v18060620/s1, Figure S1: SARS-CoV-2 (–) sgRNA and (+) sgmRNA expression during infection; Figure S2: rMHV-GFP (–) sgRNA expression correlates with TRS duplex ΔG.
