# Supplementary material for: RNA Folding Energy of Long-Range Genomic Interactions Regulates Discontinuous Transcription in SARS-CoV-2
Source: Viruses. 2026 May 29;18(6):620. doi: 10.3390/v18060620 (PMC13307601; doi:10.3390/v18060620)
Supplement: Supplementary file 1 [file viruses-18-00620-s001.zip › viruses-4320326-supplementary.pdf]

SUPPLEMENTARY FIGURES AND LEGENDS

Related to

RNA Folding Energy of Long-Range Genomic Interactions Regulates Discontinuous Transcription in SARS-CoV-2

Stephen J. Ross [1,2,3,†], Chengjin Ye [4,†], Simon Moxon [5], Elke Mühlberger [1,2], Luis Martinez-Sobrido [4] and Daniel Cifuentes [1,3,*]

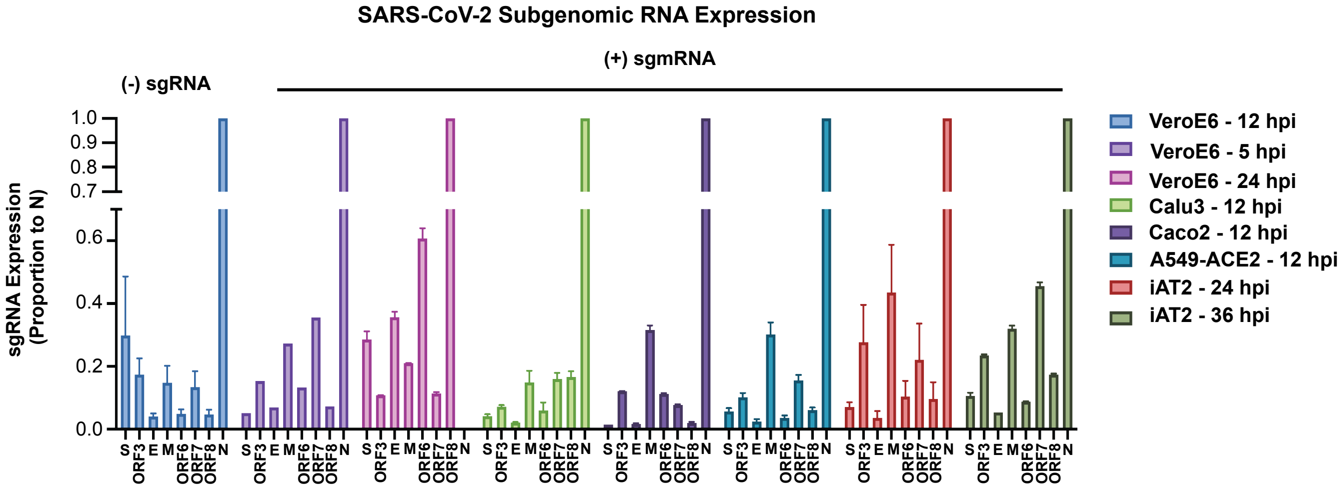

Supplementary Figure S1.

SARS-CoV-2 (–) sgRNA and (+) sgmRNA expression during infection.
1 x 106 Vero E6 cells were infected with rSARS-CoV-2 at MOI of 10. RNA was harvested 12 hpi and used for (–) sgRNA library preparation. Additionally, publicly available poly(A)-RNA or bulk RNA sequencing datasets from SARS-CoV-2 infections in cell types including Vero E6, Calu-3, Caco-2, A549-ACE2 (A549 cells expressing hACE2), and induced pluripotent stem cell-derived alveolar epithelial type 2 (iAT2) cells at distinct time points were analyzed for (+) sgmRNA expression. Expression values are normalized to the N transcript (set to 1). Values for (–) sgRNA in Vero E6 cells and (+) sgmRNA in Vero E6 and iAT2 cells at 24h are also shown in Figure 1E.

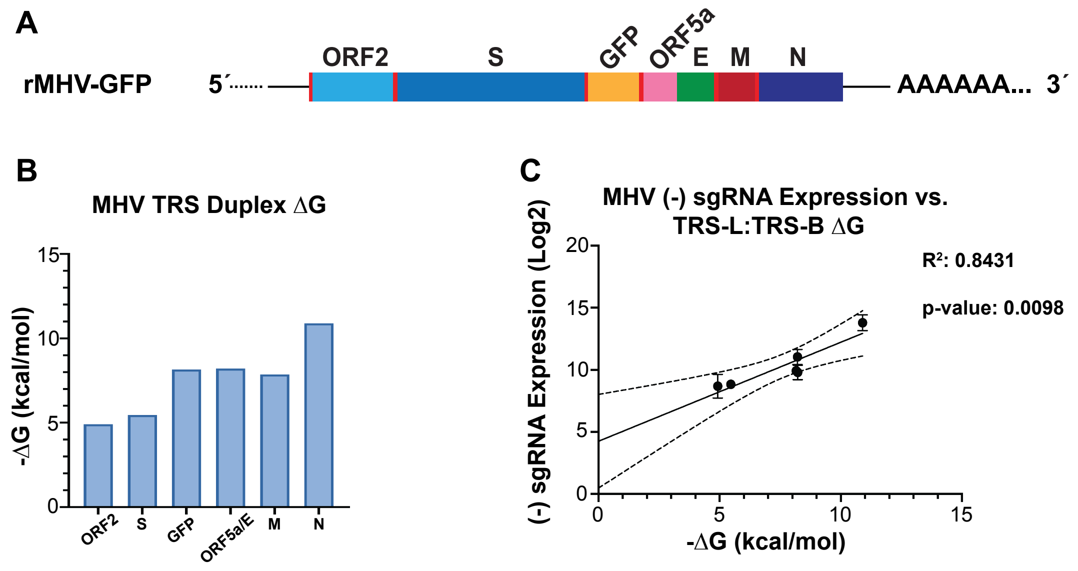

Supplementary Figure S2.

rMHV-GFP (–) sgRNA expression correlates with TRS duplex ΔG.
(A) Schematic of the rMHV structural and accessory genes. (B) rMHV TRS duplex ΔG (+10 nucleotides from TRS CS) was calculated using the ViennaRNA suite RNAcofold algorithm for each TRS-B. Values are plotted as kcal/mol. (C) rMHV TRS duplex ΔG (+10 nucleotides from TRS CS) from (B) were plotted against rMHV (–) sgRNA expression values from Figure 2B. Linear regression analysis was performed to calculate the correlation coefficient.
.
